# Combined effect of body mass index and waist-height ratio on incident diabetes; a population based cohort study

**DOI:** 10.3164/jcbn.16-116

**Published:** 2017-07-28

**Authors:** Kazuteru Mitsuhashi, Yoshitaka Hashimoto, Muhei Tanaka, Hitoshi Toda, Shinobu Matsumoto, Emi Ushigome, Mai Asano, Masahiro Yamazaki, Yohei Oda, Michiaki Fukui

**Affiliations:** 1 Department of Endocrinology and Metabolism, Kyoto Prefectural University of Medicine, Graduate School of Medical Science, 465 Kajii-cho, Kawaramachi-Hirokoji, Kamigyo-ku, Kyoto 602-8566, Japan; 2 Department of Internal Medicine, Oike Clinic, 11 Shimoai-cho, Nishinokyo, Nakagyo-ku, Kyoto, 604-8436, Japan

**Keywords:** obesity, body mass index, type 2 diabetes, abdominal obesity, waist circumference

## Abstract

We investigated the impact of combined effect of body mass index and waist-to-height ratio on risk of diabetes. Overweight and abdominal obesity were defined as body mass index ≥23 kg/m^2^ and waist-to-height ratio ≥0.5, respectively. We divided participants into four groups according to presence of overweight and/or abdominal obesity. About 20% individuals with overweight did not complicated with an abdominal obesity. Among 3,737 participants, 286 participants had diabetes at baseline-examination. Adjusted odds ratios for prevalence of diabetes compared with non-overweight participants without abdominal obesity were as follow: 1.87 (95% confidence interval 1.09–3.14, *p* = 0.024) in non-overweight participants with abdominal obesity, 1.51 (0.87–2.55, *p* = 0.141) in overweight participants without abdominal obesity and 3.25 (2.37–4.52, *p*<0.001) in overweight participants with abdominal obesity. In the follow-up examination, 86 participants were diagnosed as diabetes among 2,263 participants. Adjusted odds ratios for incident diabetes were as follow: 2.59 (0.98–6.44, *p* = 0.056) in non-overweight participants with abdominal obesity, 1.65 (0.64–4.00, *p* = 0.288) in overweight participants without abdominal obesity and 2.77 (1.55–5.15, *p*<0.001) in overweight participants with abdominal obesity. Non-overweight individuals with abdominal obesity as well as overweight individuals with abdominal obesity was associated with diabetes compared with non-overweight individuals without abdominal obesity.

## Introduction

Obesity, which is a major public health problem worldwide,^(1)^ is known as a risk of incident type 2 diabetes.^(2–5)^ Body mass index (BMI) has been used as a proxy for obesity, because it is the most economical and practical approach in both clinical and epidemiologic settings.^(6)^ On the other hand, waist circumstance is strongly correlated with abdominal fat measures from advanced imaging techniques, and thought to represent fat stored in visceral depots.^(7)^ In addition, it has been reported that waist-to-height ratio (WHtR) is useful for detecting abdominal obesity^(8)^ and that WHtR is a simple and rapid screening tool, including its ability to identify health risks in both men and women, in different ethnic groups, and in all age groups.^(5,9)^

Previous studies showed that both BMI^(2–4)^ and WHtR^(5,10–12)^ are associated with incident diabetes. However, the impact of combined effect of BMI and WHtR on incident diabetes remains to be elucidated. Therefore, we investigated the association between the combined effect of BMI and WHtR, and prevalence or incident type 2 diabetes in this population based study.

## Materials and Methods

### Participants and study design

We designed a cross-sectional study and a 5-years follow-up cohort study to investigate the impact of combined effect of BMI and WHtR on prevalence or incident diabetes in participants who received a medical health checkup program at Oike Clinic, Kyoto, Japan. The Oike Health Survey is an ongoing cohort investigation of risk factors for chronic diseases including hypertension, diabetes and chronic kidney disease.^(13)^ In Japan, yearly routine examination for employees is legally mandated and all or most of the costs for the health check-up are usually paid by their employers. The Oike Clinic provides regular health check-up for the employees of various companies. Participants, who received health check-up at Oike Clinic in 2009 were included in this study. We excluded the participants with missing data of body weight or waist circumstance and the participants who did not have a data for diabetes in the cross-sectional study. Then, we excluded the participants who were diabetes at baseline examination and the participants who did not received follow-up examination in 2014 in the retrospective cohort study. The study was conducted in accordance with Declaration of Helsinki and approval for the study was obtained from the Ethical Committee of Oike Clinic. Informed consent was obtained from each participant.

### Date collection and measurements

All participants provided details of their demographics. We classified the participants as non-smokers, ex-smokers or current-smokers according to a self-administered questionnaire. Habit of exercise was defined as performing any kind of sports at least once a week. Habit of alcohol was defined as daily alcohol consumption. Body mass index was calculated as body weight in kilograms divided by the square of the participant’s height in meters. Waist-to-height ratio was calculated by dividing the waist circumference by the participant’s height. After an overnight fast, venous blood was collected for the measurement of the levels of various factors, including fasting plasma glucose (FPG), triglycerides and high-density lipoprotein (HDL) cholesterol. Hemoglobin A1c (HbA1c) was assayed using high-performance liquid chromatography. The value for HbA1c (%) was estimated as the National Glycohemoglobin Standardization Program value (%) calculated by the formula HbA1c (%) = HbA1c (Japan Diabetes Society) (%) × 1.02 + 0.25%.^(14)^ Diagnosis of type 2 diabetes was made according to the American Diabetes Association (ADA) criteria of a FPG level of ≥7.0 mM, self-reported clinician-diagnosed diabetes or HbA1c ≥6.5% (48 mmol/mol; National Glycohemoglobin Standardization Program, NGSP).^(15)^ Participants with fasting plasma glucose ≥5.6 mM were consider to impaired fasting glucose.^(15)^

### Definition of overweight and abdominal obesity

 Body mass index ≥23.0 kg/m^2^, which has been proposed as a cut-off for the diagnosis of overweight in Asian people,^(1,16)^ was defined as overweight. This definition of overweight has often been used in Japanese population.^(17)^ In addition, Hsu *et al.*^(18)^ recommended a cut-off point of BMI 23 for the Asian American population, because this population was susceptible to overweight on incident type 2 diabetes. Abdominal obesity was defined as WHtR ≥0.5, according to the recommended criteria for diabetes.^(5)^ Then, participants were categorized at the baseline examination into four groups: 1) non-overweight participants without abdominal obesity group, 2) non-overweight participants with abdominal obesity group, 3) overweight participants without abdominal obesity group or 4) overweight participants with abdominal obesity group.

### Statistical analysis

Continuous variables were expressed as mean (SD) and categorical variables were expressed as number. Student *t* tests or χ^2^ tests was conducted to assess the statistical signiﬁcance of differences between participants without diabetes and participants with diabetes. In addition, the study participants were divided into four groups based on the presence of overweight and/or abdominal obesity and baseline characteristics of four groups were compared. The analysis of continuous variables to assess differences was determined by Tukey HSD test. The analysis of categorical variables to assess differences was determined by the χ^2^ test. Odds ratio (OR) of the four groups for prevalence or incident type 2 diabetes was calculated by logistic regression analysis. The following variables were analyzed as potential covariates: age, sex, smoking status, habit of exercise, habit of alcohol and family history of diabetes in the cross-sectional study. The following variables were analyzed as potential covariates: age, sex, smoking status, habit of exercise, habit of alcohol, family history of diabetes and impaired fasting glucose at baseline examination in the cohort study. The statistical analyses were performed using the JMP software version 10.0 software (SAS Institute Inc., Cary, NC) and *p* value <0.05 was considered to represent a statistically significant difference.

## Results

### Cross-sectional study

**Fig. 1:**
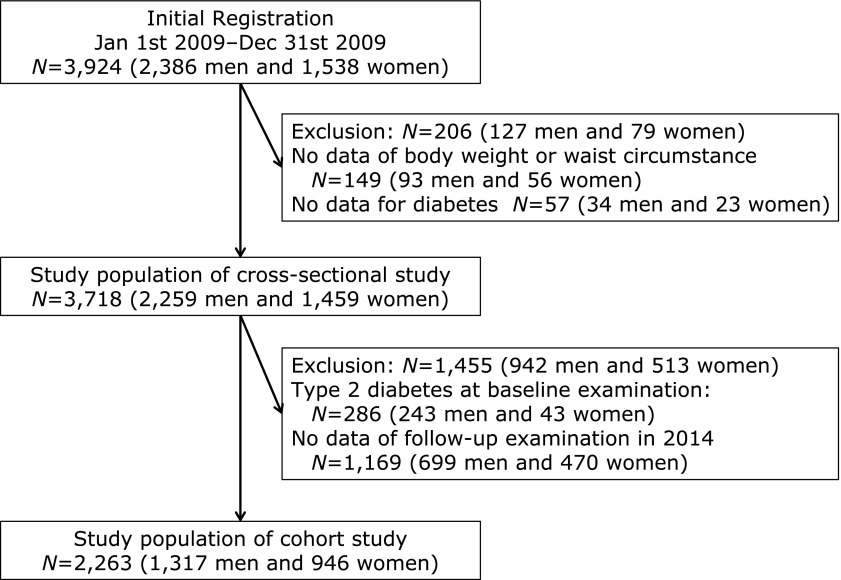
Inclusion and exclusion flow chart.

In 2009, we enrolled 3,924 participants (Fig. 1). Among them, 187 participants were excluded. Thus, 3,737 participants were eligible for the cross-sectional study. Baseline characteristics of participants are shown in Table 1. About 20% individuals with overweight did not complicated with an abdominal obesity. Moreover about 20% individuals without overweight complicated with an abdominal obesity.

At baseline examination, 286 participants have diabetes. The prevalence rate of diabetes was 3.2% (case/*n* = 58/1,795) in non-overweight participants without abdominal obesity group, 7.2% (25/345) in non-overweight participants with abdominal obesity, 5.9% (21/355) in overweight participants without abdominal obesity and 14.7% (182/1,242) in overweight participants with abdominal obesity. The adjusted ORs for prevalence of diabetes compared with non-overweight participants without abdominal obesity were as follow: 1.87 (95% confidence interval (CI) 1.09–3.14, *p* = 0.024] in non-overweight participants with abdominal obesity, 1.51 (95% CI 0.87–2.55, *p* = 0.141) in overweight participants without abdominal obesity and 3.25 (95% CI 2.37–4.52, *p*<0.001) in overweight participants with abdominal obesity (Table 2).

### Retrospective cohort study

In the cohort study, we excluded the participants who were diabetes at baseline examination and the participants who did not received follow-up examination, which performed in 2014 (Fig. 1). Thus, 2,263 participants were eligible for the cohort study. Baseline characteristics of participants of cohort study according to four groups are shown in Table 3. Participants with abdominal obesity had higher blood pressure, FPG and triglycerides compared with participants without abdominal obesity. In addition, overweight participants with abdominal obesity had higher blood pressure, FPG and triglycerides, and lower HDL cholesterol compared with non-overweight participants with abdominal obesity.

At follow-up examination, 86 participants have diabetes. The incident rate of diabetes was 1.5% (case/*n* = 17/1,138) in non-overweight participants without abdominal obesity, 4.4% (8/183) in non-overweight participants with abdominal obesity, 3.4% (8/235) in overweight participants without abdominal obesity and 7.5% (53/707) in overweight participants with abdominal obesity. The adjusted ORs for incident diabetes compared with non-overweight participants without abdominal obesity were as follow: 2.59 (95% CI 0.98–6.44, *p* = 0.056) in non-overweight participants with abdominal obesity, 1.65 (95% CI 0.64–4.00, *p* = 0.283) in overweight participants without abdominal obesity and 2.77 (95% CI 1.55–5.15, *p*<0.001) in overweight participants with abdominal obesity (Table 4).

## Discussion

In this study, we showed that overweight individuals with abdominal obesity was associated with higher risk of prevalence or incident diabetes compared with non-overweight individuals without abdominal obesity. Furthermore, non-overweight individuals with abdominal obesity was also associated with higher risk of prevalence or incident diabetes compared with non-overweight individuals without abdominal obesity in our study population. These results revealed that abdominal obesity, an important component of metabolic syndrome, may be considered high risk obesity for diabetes.

Possible explanations for the association between combination of overweight and abdominal obesity and risk of diabetes are as follows. It is well known that obesity is associated with insulin resistance.^(19)^ B-cell exhaustion due to continuous insulin resistance leads to decline of insulin secretion.^(20)^ Because East Asian people have a limited innate capacity of insulin secretion,^(21)^ even a small increase of insulin resistance might lead to incident type 2 diabetes. On the other hand, it has been reported that visceral adiposity, which has closely association with abdominal obesity,^(22)^ is the independent risk factor for insulin resistance.^(23)^ It has been reported that advanced glycation end products, which has a close association with visceral adiposity, is associated with diabetes and diabetes complications.^(24)^ In addition, abdominal obesity is more related to diabetes than overall obesity.^(10,11)^ Recent studies revealed that a subset of individuals with obesity who have a low burden of adiposity-related metabolic abnormalities compared with individuals with ‘at risk’ obesity, the so-called ‘metabolically healthy obesity’ (MHO) phenotype.^(25–27)^ Visceral adiposity of MHO is lower than metabolically abnormal obesity.^(28)^ Furthermore, we previously reported that overweight individuals with non-alcoholic fatty liver disease (NAFLD) and non-overweight individuals with NAFLD is associated with incident diabetes.^(17)^ NAFLD is caused by ectopic fat accumulation in the liver.^(29)^ Ectopic fat accumulation, which has a close association with visceral adiposity, is reported to be strongly correlated with insulin resistance.^(30)^ Unfortunately, we did not have data of the presence of NAFLD in this study population. Thus, the concept of metabolically abnormal or presence of NAFLD is almost same as presence of abdominal obesity. In fact, the result of this study was the same as past studies of MHO phenotype^(26,27)^ or presence of NAFLD.^(17)^ Taking these finding together, non-overweight individuals with abdominal obesity as well as overweight individuals with abdominal obesity is associated with higher risk of diabetes compared with non-overweight individuals without abdominal obesity.

Dietary modification is an important for the prevention of incident type 2 diabetes. In fact, it has been reported that supplementation of soy isoflavones is effective for improving lipid profiles and apolipoprotein levels in patients with type 2 diabetes.^(31)^

Some limitations of our study should be noted. First, body fat distribution was assessed based on anthropometric indicators alone in this study. However, WHtR has been reported to be useful for central fat distribution.^(32)^ Second, we did not have a data of insulin, thus, we could not assess the insulin resistance. Finally, the study population consisted of Japanese men and women, therefore, it is uncertain whether these findings are generalized in other ethnic groups.

In conclusion, our study firstly showed an evidence that the risk of incident diabetes in overweight individuals with abdominal obesity was significantly higher than that in non-overweight individuals without abdominal obesity. In addition, the risk of incident diabetes in non-overweight individuals with abdominal obesity was also higher than that in non-overweight individuals without abdominal obesity.

## Figures and Tables

**Table 1:** Characteristics of study participants of cross-sectional study

	Participants without diabetes	Participants with diabetes	*p* value
*N*	3,432	286	—
Age (years)	55.1 (10.2)	63.2 (9.0)	<0.001
Sex (male/female)	2,016/1,416	243/43	<0.001
Body mass index (kg/m^2^)	22.5 (3.0)	24.9 (3.4)	<0.001
Waist circumstance (cm)	80.6 (9.0)	88.1 (8.8)	<0.001
Systolic blood pressure (mmHg)	121.0 (15.3)	129.3 (14.1)	<0.001
Diastolic blood pressure (mmHg)	75.7 (10.4)	79.4 (9.5)	<0.001
Fasting plasma glucose (mM)	5.1 (0.5)	7.6 (1.7)	<0.001
HbA1c (%)	5.4 (0.4)	6.6 (0.9)	<0.001
HbA1c (mmol/mol)	34 (5)	48 (10)	<0.001
Total cholesterol (mM)	5.4 (0.0)	5.2 (0.1)	<0.001
Triglycerides (mM)	1.2 (0.8)	1.8 (1.5)	<0.001
HDL cholesterol (mM)	1.7 (0.5)	1.4 (0.4)	<0.001
Smoking (non-/ex-/current-)	2,444/439/548	181/50/55	0.014
Habit of alcohol (–/+)	2,158/1,272	177/109	0.73
Habit of exercise (–/+)	1,398/2,033	97/189	0.024
Family history of diabetes (–/+)	2,773/639	164/122	<0.001
Overweight/abdominal obesity (–/–)/(–/+)/(+/–)/(+/+)	1,727/319/333/1,053	58/25/21/182	<0.001

HDL; high-density lipoprotein. Data are number or mean (standard deviation). Student *t* tests or χ^2^ tests was conducted to assess the statistical significance of differences between participants without diabetes and participants with diabetes.

**Table 2:** Odds ratios for prevalence of type 2 diabetes at baseline examination according to presence of overweight and/or abdominal obesity

	Case of diabetes	Model 1	*p* value	Model 2	*p* value
Non-overweight without abdominal obesity	58/1,795	1 (Reference)	—	1 (Reference)	—
Non-overweight with abdominal obesity	25/345	1.93 (1.14–3.19)	0.015	1.87 (1.09–3.14)	0.024
Overweight without abdominal obesity	21/355	1.65 (0.95–2.74)	0.072	1.51 (0.87–2.55)	0.141
Overweight with abdominal obesity	182/1,242	3.33 (2.44–4.60)	<0.001	3.25 (2.37–4.52)	<0.001
Age (per one year)	—	1.07 (1.05–1.08)	<0.001	1.08 (1.06–1.10)	<0.001
Men	—	3.26 (2.32–4.68)	<0.001	3.87 (2.67–5.70)	<0.001
Habit of alcohol	—	—	—	0.67 (0.50–0.88)	0.042
Habit of exercise	—	—	—	1.07 (0.81–1.42)	0.656
Ex-smoker	—	—	—	1.18 (0.81–1.69)	0.387
Current smoker	—	—	—	1.45 (1.01–2.07)	0.044
Family history of diabetes	—	—	—	4.31 (3.26–5.70)	<0.001

Model 1 adjusted for age and sex. Model 2 adjusted for Model 1 and habit of alcohol, habit of exercise, smoking status, and family history of diabetes.

**Table 3:** Characteristics of study participants of cohort study at the baseline examination

	Non-overweight without abdominal obesity	Non-overweight with abdominal obesity	Overweight without abdominal obesity	Overweight with abdominal obesity
*N*	1,138	183	235	707
Age (years)	52.8 (9.3)	58.8 (9.7)*	51.0 (8.9)*^,^^†^	57.1 (10.4)*^,^^‡^
Sex (male/female)^§^	556/582	44/139	203/32	514/193
Body mass index (kg/m^2^)	20.3 (1.7)	21.8 (1.0)*	24.0 (0.8)*^,^^†^	25.9 (2.2)*^,^^†^^,^^‡^
Waist circumstance (cm)	74.1 (5.9)	82.4 (3.9)*	82.1 (3.7)*	90.0 (5.9)*^,^^†^^,^^‡^
Systolic blood pressure (mmHg)	115.0 (14.4)	121.1 (14.7)*	122.5 (12.3)*	128.9 (13.8)*^,^^†^^,^^‡^
Diastolic blood pressure (mmHg)	72.0 (10.1)	75.0 (10.6)*	77.5 (8.5)*	80.8 (9.2)*^,^^†^^,^^‡^
Fasting plasma glucose (mM)	5.0 (0.5)	5.1 (0.5)*	5.1 (0.5)*	5.3 (0.5)*^,^^†^^,^^‡^
HbA1c (%)	5.3 (0.3)	5.4 (0.4)*	5.3 (0.4)	5.4 (0.4)*^,^^‡^
HbA1c (mmol/mol)	34 (4)	36 (4)*	34 (5)	36 (4)*^,^^‡^
Total cholesterol (mM)	5.3 (0.9)	5.5 (0.8)	5.3 (0.8)	5.3 (0.8)
Triglycerides (mM)	1.0 (0.6)	1.2 (0.6)*	1.3 (0.7)*	1.5 (1.0)*^,^^†^^,^^‡^
HDL cholesterol (mM)	1.9 (0.5)	1.8 (0.4)	1.6 (0.4)*^,^^†^	1.5 (0.4)*^,^^†^
Smoking (non-/ex-/current-)^§^	861/115/161	158/10/15	135/53/47	473/116/118
Habit of alcohol (–/+)^§^	754/383	128/55	137/98	419/287
Habit of exercise (–/+)^§^	449/688	82/101	68/167	287/420
Family history of diabetes (–/+)	931/207	153/30	187/48	556/151

HDL; high-density lipoprotein. Data are number or mean (standard deviation). The analyses of continuous among four groups were performed by Tukey HSD test. *vs Non-overweight without abdominal obesity, ^†^vs Non-overweight with abdominal obesity, ^‡^vs Overweight without abdominal obesity. The analyses of categorical variables among four groups were performed by χ^2^ test. ^§^*p*<0.05.

**Table 4:** Odds ratios for incident type 2 diabetes at 5 years after the baseline examination according to presence of overweight and/or abdominal obesity

	Case of diabetes	Model 1	*p* value	Model 2	*p* value	Model 3	*p* value
Non-overweight without abdominal obesity	17/1,138	1 (Reference)	—	1 (Reference)	—	1 (Reference)	—
Non-overweight with abdominal obesity	8/183	3.48 (1.37–8.21)	0.01	3.60 (1.41–8.52)	0.009	2.59 (0.98–6.44)	0.056
Overweight without abdominal obesity	8/235	1.82 (0.73–4.20)	0.192	1.72 (0.68–3.99)	0.239	1.65 (0.64–4.00)	0.288
Overweight with abdominal obesity	53/707	4.13 (2.38–7.49)	<0.001	3.95 (2.28–7.17)	<0.001	2.77 (1.55–5.15)	<0.001
Age (per one year)		1.02 (1.00–1.04)	0.097	1.03 (1.01–1.05)	0.01	1.01 (0.99–1.04)	0.335
Men	—	2.54 (1.47–4.66)	<0.001	2.59 (1.43–4.90)	0.001	1.69 (0.91–3.26)	0.1
Habit of alcohol	—	—	—	0.64 (0.39–1.03)	0.068	0.58 (0.34–0.96)	0.033
Habit of exercise	—	—	—	0.90 (0.57–1.44)	0.659	0.90 (0.55–1.48)	0.664
Ex-smoker	—	—	—	1.66 (0.89–3.00)	0.111	1.61 (0.82–3.05)	0.164
Current smoker	—	—	—	2.30 (1.29–4.02)	0.005	2.29 (1.22–4.23)	0.01
Family history of diabetes	—	—	—	1.95 (1.16–3.19)	0.013	1.40 (0.80–2.38)	0.233
Impaired fasting glucose	—	—	—	—	—	20.0 (11.4–37.2)	<0.001

Model 1 adjusted for age and sex. Model 2 adjusted for Model 1 and habit of alcohol, habit of exercise, smoking status, and family history of diabetes. Model 3 adjusted for Model 2 and impaired fasting glucose at baseline examination.

## Abbreviations

BMI: body mass index
FPG: fasting plasma glucose
HbA1c: hemoglobin A1c
HDL: high-density lipoprotein
NAFLD: non-alcoholic fatty liver disease
MHO: metabolically healthy obesity
WHtR: waist-to-height ratio
